# Application of BMP-2 and its gene delivery vehicles in dentistry

**DOI:** 10.1016/j.sdentj.2024.03.015

**Published:** 2024-03-22

**Authors:** Valeriya Sergeevna Kuznetsova, Andrey Vyacheslavovich Vasilyev, Tatiana Borisovna Bukharova, Irina Alekseevna Nedorubova, Dmitry Vadimovich Goldshtein, Vladimir Karpovich Popov, Anatoly Alekseevich Kulakov

**Affiliations:** a Central Research Institute of Dentistry and Maxillofacial Surgery, Moscow, Russia; b Research Centre for Medical Genetics, Moscow, Russia; c Federal Scientific Research Centre “Crystallography and Photonics”, Russian Academy of Sciences, Moscow, Russia

**Keywords:** Bone morphogenetic protein, BMP-2, Gene-activated materials, Osteogenesis

## Abstract

The restoration of bone defects resulting from tooth loss, periodontal disease, severe trauma, tumour resection and congenital malformations is a crucial task in dentistry and maxillofacial surgery. Growth factor- and gene-activated bone graft substitutes can be used instead of traditional materials to solve these problems. New materials will overcome the low efficacy and difficulties associated with the use of traditional bone substitutes in complex situations. One of the most well-studied active components for bone graft substitutes is bone morphogenetic protein-2 (BMP-2), which has strong osteoinductive properties. The aim of this review was to examine the use of BMP-2 protein and gene therapy for bone regeneration in the oral and maxillofacial region and to discuss its future use.

## 1 Introduction

Orthopaedics, neurosurgery and maxillofacial surgery are the main areas in which bone graft materials are used worldwide. The most intensive growth in the consumption of these materials is in dentistry due to bone grafting associated with the placement of dental implants and treatment of periodontal diseases (Cha et al., 2016, Zhang et al., 2022, Allied Market Research, xxxx). Although traditional bone graft materials and substitutes are still widely used, they are not sufficiently effective, cannot be used to repair extensive bone defects and lead to the formation of fibrosis. Therefore, researchers continue to develop and introduce into clinical practice more effective materials consisting of a scaffold carrying bioactive components: cells (tissue-engineered constructs), growth factors or genetic vectors encoding osteoinductive proteins to facilitate complete bone regeneration (Szwed-Georgiou et al., 2023). Compared to autogenous bone tissue, the use of these materials eliminates the need for the patient’s own tissue and the formation of donor sites and reduces the trauma, pain, risk of bleeding and associated postoperative complications (Nkenke and Neukam, 2014, Jensen et al., 2016, Drăgan and Nemţoi, 2022).

**Fig. 1:**
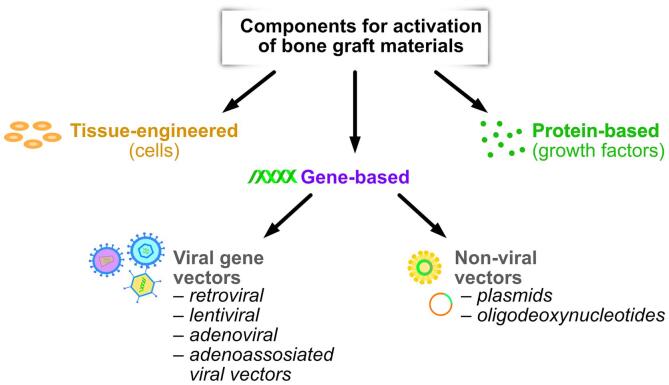
Activating components for bone graft materials.

Tissue-engineered constructs are not suitable for restoring extensive bone defects because insufficient vascular sprouting leads to poor blood supply and the death of the transplanted cells (Menger et al., 2022). Further, the implantation of allogeneic and xenogeneic stem cells can lead to an immune-mediated inflammatory response (Baranovskii et al., 2022). The production, storage and registration of such products are also difficult (Bozo et al., 2021). More promising approaches to bone regeneration include the use of materials containing osteoinductive components – growth factors or vectors carrying its genes (Fig. 1). Bone morphogenetic protein-2 (BMP-2) is the most well-researched osteoinductive protein, a member of BMP family (Kang et al., 2004).

In this article, we will review the application of BMP-2 protein and gene therapy for bone tissue regeneration in dentistry and maxillofacial surgery and discuss the use of activated materials in the dental field in the future.

## 2 Methods

A review of the relevant scientific literature on the use of BMP-2 and its gene delivery vehicles in dentistry was conducted in January 2023 using Google Scholar and PubMed. Publications included reviews, book chapters, original research papers, *in vitro* experiments, *in vivo* animal or human studies and clinical trials. From the search results, articles were selected that showed or discussed results of the use of protein or gene therapy with BMP-2 for bone regeneration in the maxillofacial region. The key words were ‘bone morphogenetic protein’, ‘rhBMP-2’, ‘BMP2’, ‘dentistry’, ‘maxillofacial’, ‘alveolar ridge augmentation’ and ‘sinus floor augmentation’. Articles on related topics were reviewed as well. The papers cited in the journals were also reviewed for relevance and included. Full access to the document was a prerequisite for inclusion.

## 3 Protein and gene therapy for bone tissue regeneration

### 3.1 Protein-activated materials

Proteins are relatively easy to produce in large quantities and can be used to manufacture materials with less batch-to-batch variation. *In vitro* and *in vivo* studies, along with clinical trials, have demonstrated the high efficacy of recombinant human BMP-2-containing materials in maxillofacial bone regeneration (Han et al., 2022, Ho et al., 2010, Torrecillas-Martinez et al., 2013). However, studies have also shown the disadvantages of these materials (Kuznetsova et al., 2019). They are mainly associated with the use of supraphysiological doses of protein, which leads to complications in the postoperative period (James et al., 2016). The use of gene vectors can solve these problems by providing local production of the necessary amount of osteoinductive protein for bone tissue regeneration.

### 3.2 Gene-activated materials

Gene therapy is performed in two steps. The first non-specific step involves the release of nucleic acids from carriers, entry into the cell and their translocation into the nucleus, followed by transcription, translation and protein production. The synthesised proteins influence the surrounding cells and cause them to differentiate in the next specific step (Barhate et al., 2023). There are *in vivo* and *ex vivo* types of gene therapy. Nucleic acids can be delivered into cells by viral or non-viral vectors.

#### 3.2.1 Types of gene therapy

*In vivo* the therapeutic gene is injected directly into the patient’s body. It is simple and minimally invasive and does not involve high financial and time costs (Siddique et al., 2016) Fig. 2. But it tends to be less effective and less predictable than the *ex vivo* approach (Barhate et al., 2023). The *ex vivo* method is more complex and expensive. It requires isolation of cells from the biopsy specimen, expansion in cell culture, modification *ex vivo* and subsequent implantation of the cells into the bone defect area. The major advantages of the *ex vivo* method are the absence of immune reactions to the introduction of autologous cells and the ability to control the penetration efficiency of genetic constructs or DNA complexes into the target cells (Kumar et al., 2016). Disadvantages include the additional procedure of tissue extraction and time cost, which limit its use in acute conditions (Park et al., 2019). For local application, *in vivo* gene therapy is preferred, as it does not require autologous cell harvesting and is less labour intensive (De la Vega et al., 2021).

**Fig. 2:**
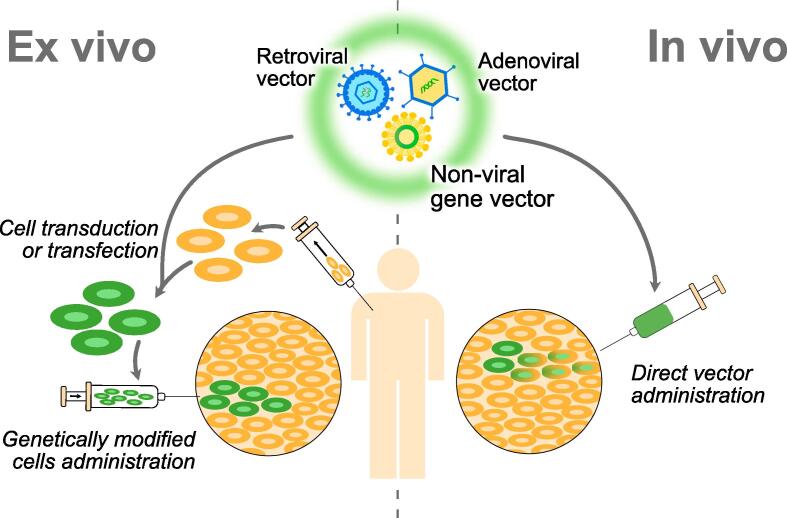
Strategies of *in vivo* and ex vivo gene therapy.

#### 3.2.2 Viral vectors

**Fig. 3:**
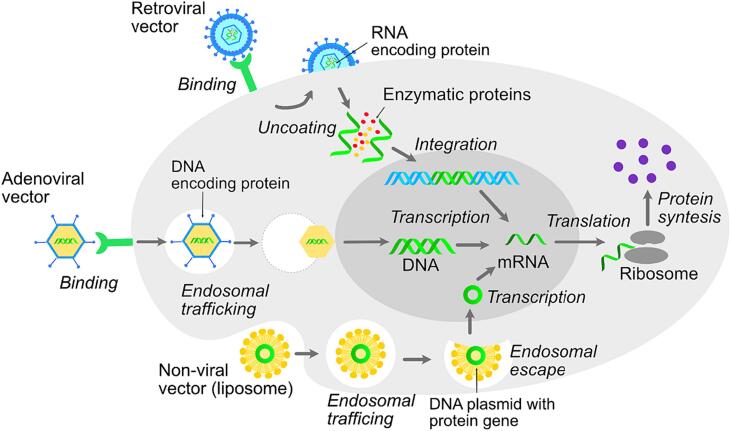
Scheme of viral and non-viral vectors action.

The use of viruses to deliver genetic constructs is based on their natural ability to transfer genetic material into cells (Fig. 3). Adenoviral, adeno-associated, retroviral and lentiviral vectors are the most commonly used for gene delivery (Barhate et al., 2023).

Retroviruses were the first vectors used in *in vivo* gene therapy trials. They have a high packaging capacity – the ability to carry long genetic sequences encoding the required proteins – and long-term gene expression resulting from integration into the host genome. Disadvantages of retroviral vectors include a limited ability to transduce only dividing cells and a high risk of insertional mutagenesis – the process of altering the patient’s DNA sequence by inserting fragments of foreign genetic information into the genome (Pensak and Lieberman, 2013, Vakhshori et al., 2018).

Lentiviruses are a subgroup of retroviruses. Compared to other types of retroviral vectors, lentiviruses have a larger packaging capacity and can infect non-dividing or terminally differentiated cells. They also carry a risk of insertional mutagenesis that limit their use for gene delivery (Helio et al., 2013).

Adenoviruses and adeno-associated viruses are the most commonly used vectors, as they can infect both dividing and non-dividing cells. Moreover, they have a much lower risk of insertional mutagenesis because they do not directly enter the nucleus and remain in the cytoplasm (Lee et al., 2017). Adeno-associated viral vectors have low immunogenicity and high transduction efficiency, and they are non-pathogenic (Shirley et al., 2020). A disadvantage of adenoviral vectors is a humoral response that prevents reactivation of transgene expression after repeated administration (Zhang et al., 2023).

Adenoviral vectors are more commonly used for *ex vivo* delivery of BMP-2. This is related with high efficacy of the *ex vivo* method compared to the *in vivo* method*.* In the calvarial critical size model, the efficiency of neoosteogenesis and the volume fraction of newly formed bone tissue are higher with *ex vivo* implantation (33 %) than *in vivo* implantation (28 %) (Bukharova et al., 2023).

The possibility of adverse effects like systemic inflammatory reactions and insertional mutagenesis limits viral vectors active use in clinical practice (Zhao et al., 2022). In addition, the repeated application can lead to the production of antibodies, which prevents the successful delivery of viral vectors and reduces the efficiency of transduction (Shirley et al., 2020). To overcome some of these challenges, non-viral delivery methods have been developed (Hardee et al., 2017).

#### 3.2.3 Non-viral vectors

Non-viral vectors include vectors based on plasmids or oligodeoxynucleotides. Plasmids are extrachromosomal, autonomously replicating, double-stranded circular DNA molecules found in bacteria that control fertility, drug resistance and the degradation of various compounds. Compared to viral vectors, they have a low immunogenicity and high packaging capacity, lower risk of systemic complications. They are also easier and cheaper to produce (Loh and Lee, 2012, Hardee et al., 2017). Compared to viral vectors they show lower delivery efficiency. The presence of bacterial sequences can lead to gene silencing or a strong immune response (Ahmad-Nejad et al., 2002, Faurez et al., 2010, Lu et al., 2012). Because plasmids remain in the cell as non-replicating DNA rings (episomes), the expression of the transgenes they carry is transient and dependent on cell-division processes (Hardee et al., 2017).

Various physical and chemical methods are used to improve the efficiency of plasmid delivery

**Physical methods** act locally on cellular barriers. They increase membrane permeability and force cells to accept exogenous nucleic acid molecules. The most common physical delivery methods are electroporation and sonoporation.

Electroporation involves the delivery of short electrical pulses that destabilise the cell membrane (Raisin et al., 2016), while in sonoporation the transgene penetrates the cell under the influence of high-intensity ultrasound waves. Direct action on cells by physical methods makes techniques difficult to use *in vivo*. Hence, they are mainly used the cellular delivery of gene constructs *ex vivo*.

**Chemical methods** include the use of inorganic nanoparticles, lipid-based systems and polymers.

The advantages of nanoparticles use are the ability to transfect post-mitotic cells *in vivo* and *in vitro*, the absence of immune reactions and the ease of modification, which makes it possible to control the absorption and release of nucleic acids (Al-Dosari and Gao, 2009, Jin et al., 2022).

Cationic liposomes are structures made up of positively charged lipids. They protect nucleic acids from extracellular or intracellular nucleases and ensure penetration through the cell membrane (Tassler et al., 2019). Cationic polymers neutralise the charge of DNA and condense nucleic acids, protecting them from nucleases, facilitating the uptake of DNA complexes into cells and aiding their endosomal release. Both natural (chitosan, alginate, gelatin, cationic cellulose and cyclodextrin) and synthetic polymers (polyethyleneimines, polyamines, polypropyleneimines, etc.) are used for nucleic acid delivery. Cationic polymers and liposomes have low toxicity and can be degraded by cellular enzymes (Cai et al., 2023).

Comparison of the efficiency of cationic lipids, cationic polymers and inorganic particles showed that, depending on the cell line, lipofection was five to more than 100 times more effective than transfection with calcium phosphate or diethylaminoethyl-dextran transfection (Kulkarni et al., 2018). Other studies have also shown the promise of lipoplexes compared to nucleic acid delivery using inorganic particles and polymers (Nedorubova et al., 2021, Yang et al., 2008).

## 4 Bone graft materials with BMP-2 in dentistry and maxillofacial surgery

### 4.1 Protein-activated materials

Over the last 15 years, protein-activated bone graft materials with BMP-2 have been developed and introduced into clinical practice (Kuznetsova et al., 2019). The first Infuse Bone Graft (Medtronic, USA) was approved in 2007 for sinus lift elevation and alveolar ridge augmentation. Since then, materials containing BMP-2 have been actively used in bone-grafting procedures, and their efficacy has been reported (Table 1). A multicentre prospective clinical trial showed that after sinus floor elevation and dental implantation, new bone volume and implant placement success rates were similar for BMP-2 and autogenous bone graft groups (Triplett et al., 2009). However, the results of other studies have been less positive. For example, the use of BMP-2 with allografts led to an increase in bone height, but the new bone volume was less than in the control groups using traditional materials alone (Kao et al., 2012, Kelly et al., 2016). This may be related to the high affinity for the demineralised bone matrix, which contributes to the retention of BMP-2 and limits its effect (Vasilyev et al., 2020). A systematic review and meta-analysis of alveolar ridge augmentation using BMP-2 also showed that it influences the amount of new bone. However, no additional clinical benefits were shown compared with other bone graft materials (Kelly et al., 2016).

**Table 1:** Application of BMP-2-activated bone graft materials and their future development.

Commercial bone graft material	Components	Indications for dentistry and maxillofacial surgery
Protein-activated therapy		
*Clinical data*		
‘Infuse Bone Graft’ (Medtronic, USA);	Absorbable collagen sponge, a vial with lyophilised rhBMP-2, a vial with sterile water for reconstituting rhBMP-2	FDA approved: - alveolar ridge augmentation (Fiorellini et al., 2005, Howell et al., 1997), - sinus floor elevation (Boyne et al., 2005, Boyne et al., 1997) Off-label: - alveolar cleft reconstruction (Alonso et al., 2010, Canan et al., 2012, Carstens et al., 2005, Chin et al., 2005, Dickinson et al., 2008, Herford et al., 2007a); - mandibular bone defect (Carter et al., 2008)
‘Novosis’ (Bongros/BMP-2) (CGBio Co., Korea)	Porous beta-tricalcium phosphate-based ceramics, a vial with lyophilised rhBMP-2, a vial with sterile water for reconstituting the rhBMP-2	- regeneration of alveolar bone tissue (Han et al., 2022, Kim et al., 2015) - maxillary sinus floor augmentation (Han et al., 2021, Kim et al., 2017)
‘Cowell BMP’ (Busan, Korea)	Biphasic calcium phosphate with freeze-dried recombinant human bone morphogenic protein type 2 (rhBMP-2) on the surface	- severely resorbed alveolar ridges, - tooth extraction socket (Huh et al., 2011), - alveolar bone loss; maxillary sinus bone loss (Kim et al., 2015), -maxillofacial reconstruction
‘Gutai’ (Shanghai Rebone Biomaterials Co., Ltd., Shanghai, China)	BMP-2-incorporated calcium phosphate cement	- alveolar cleft reconstruction (Shen et al., 2022), - regeneration of alveolar bone tissue, - maxillary sinus floor augmentation,

*Experimental data*		
The main technology improvements of protein therapy with BMP-2 are: - manufacture of carrier materials for controlled protein release: granules impregnated with BMP-2 (Pelletier et al., 2014; Vasilyev et al., 2020b), hydrogels (Gultian et al., 2022, Vasilyev et al., 2019); - creation of more convenient application materials: curable (Vasilyev et al., 2021), 3D printed (Bi et al., 2023); - improvement of protein production technologies (Oliveira et al., 2021); - use of heterodimeric BMP (Liu et al., 2023)		

Gene-activated therapy		
*Clinical data*		
Gene-activated materials with BMP2 are still not approved for clinical use		
*Experimental data*		
Developed materials are used in experiments for the following: - alveolar ridge augmentation (Kawai et al., 2018); - sinus floor elevation (Jhin et al., 2013, Jiang et al., 2009, Xia et al., 2011); - treatment of *peri*-implantitis (Li et al., 2017, Park et al., 2015, Xu et al., 2016) The main directions in the improvement of viral gene delivery technology are: - manufacture of vectors with long-and short-term safety: the use of non-human (Chen et al., 2009) and self-inactivating viruses (Alaee et al., 2014); - producing of viral vectors with high efficiency: self-complimentary vectors (Yazici et al., 2011), chimeric vectors (Olmsted-Davis et al., 2002).		
The main directions in the improvement of non-viral gene delivery technology are: - promotion of enzymatic protection and enhanced physical stability: the use of polymeric carriers (Rose et al., 2012) and protective copolymers (Schwabe et al., 2012); - superior delivery efficiency: the use of various transfection agents (Jalal and Dixon, 2020); - optimisation of the BMP-2 gene sequence (Hacobian et al., 2016), gene cassette (Zimmermann et al., 2020).		

In addition to applications approved by the Food and Drug Administration (FDA) in the US, materials with BMP-2 are used for alveolar cleft reconstruction (Canan et al., 2012, Hammoudeh et al., 2017, Makar et al., 2021), the treatment of medication-related osteonecrosis of the jaw (Min et al., 2020, Park et al., 2017), maxillofacial trauma (Herford, 2017) and mandibular reconstruction after tumor removal (Cicciù et al., 2014, Zétola et al., 2011). Studies have shown that the use of BMP-2 in these complex cases is reasonable and has advantages over traditional bone graft substitutes. It can reduce donor site morbidity, prevent the need for a second operation in patients with alveolar clefts and reduce the consolidation period of fracture healing (Canan et al., 2012, Dickinson et al., 2008, Herford et al., 2007b). Despite these benefits, there are risks associated with the off-label use of BMP-2. The main side effect of BMP-2 use in dentistry and maxillofacial surgery is massive long-term oedema of the soft tissues associated with inflammatory reactions. Ectopic osteogenesis is also a concern for specialists. The risk of ectopic osteogenesis is increased by the use of supraphysiological protein concentrations in scaffolds, which cannot withstand the pressure of the surrounding tissue (James et al., 2016). In an *in vivo* experiment in which dental implants were placed in the jaws of pigs using high concentrations of BMP-2, bone formation was too intense, causing previously placed implants to change position and become displaced from the bone (Wikesjö et al., 2008). Ectopic osteogenesis is a rare complication in dental and maxillofacial surgery. Both *in vitro* and *in vivo* studies have shown a pro-oncogenic effect of the protein (Skovrlj et al., 2015). However, clinical trials of BMP-2 materials found no evidence of de novo tumor formation, and a meta-analysis could not confirm an increased risk of cancer after protein use (Wijaya et al., 2023). The disadvantages described above significantly impact the direction of further protein therapy development (Table 1).

### 4.2 Gene-activated materials

The introduction of gene-activated bone graft materials into clinical practice is complicated. To date, there have been only two clinical trials using gene therapy for bone regeneration worldwide. An open-label, non-randomised clinical trial involving 20 patients with alveolar atrophy, chronic periodontitis, periapical lesions and *peri*-implantitis showed that the use of a material containing vascular endothelial growth factor (VEGF) plasmid was effective in 100 % of patients. A long-term follow-up (approximately 30 months) study confirmed the safety and efficacy of the material, as no patients experienced failure of implant integration or *peri*-implantitis (Bozo et al., 2021). In 2019, Histograft (Histograft Ltd., Russia) became the first gene-activated material registered for bone regeneration in Russia and the world (Bozo et al., 2021). However, despite the manufacturer’s claims, VEGF plasmids do not have a direct osteoinductive effect. They only stimulate vascular growth.

Such results offer hope for further implementation of gene therapy in clinical practice. *In vivo* studies have shown the promise of BMP2 gene therapy for a number of indications: alveolar ridge augmentation (Kawai et al., 2018), sinus floor elevation (Jhin et al., 2013, Jiang et al., 2009, Xia et al., 2011) and the treatment of *peri*-implantitis (Li et al., 2017, Park et al., 2015, Xu et al., 2016). However, the long-term efficacy and safety of the materials still need to be investigated.

The use of gene-activated materials is associated with the reduction of the local osteoinductive protein amount while maintaining its efficacy through long-term production by cells. However, the expression of viral envelope proteins can lead to inflammation in the transduced tissue and significant loss of vector genomes (Boutin et al., 2010, Liu and Muruve, 2003). For non-viral vectors, the immune response is determined by the nature of the carrier of the genetic information (Judge et al., 2006). The possibility of pro-oncogenic effects of gene-activated materials is a concern, as cases of serious complications have been reported in studies *in vivo* and clinical trials of some gene therapy products (Miller et al., 2005, Nakai et al., 2005, Nguyen et al., 2021). The possibility of ectopic bone formation has not been sufficiently investigated. Local implantation of gene-activated materials limits the spread of vectors throughout the body. However, an *in vivo* study showed that ectopic bone foci can be detected in the tissue surrounding the augmented bone defect (Baltzer et al., 1999).

Table 1 summarises the studies related to the future development of protein and gene delivery technologies using BMP-2. Solving the problems related to safety and efficacy could make the use of gene therapy more promising compared to protein therapy. The synthesis of BMP-2 in cells avoids the side effects associated with the use of supraphysiological doses of osteoinductive protein.

Further, gene-activated materials are not limited to applications in areas where exogenous protein is used. Their high physical and chemical stability allows them to be used in the 3D printing of implants for the replacement of extensive discontinuous bone defects in maxillofacial surgery, and research is already being carried out on this possibility (Khvorostina et al., 2022) Fig. 4.

**Fig. 4:**
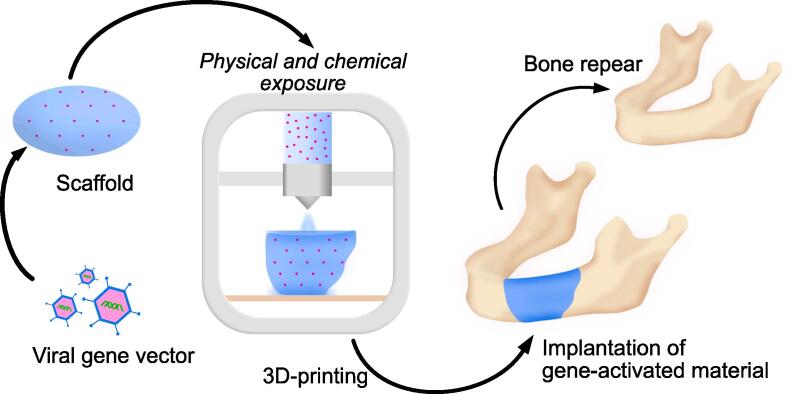
Use of gene-activated material for 3D-printed implant in extensive bone defect restoration.

## 5 Conclusion

In preclinical and clinical studies, protein- and gene-activated scaffolds have demonstrated efficacy comparable to or exceeding that of traditional materials. While the development of activated bone graft materials has undergone significant progress and advancement, the number of products approved by regulatory authorities is extremely low. This may be due to the novelty of the technologies, social, economic, bioethical and legal reasons as well as the inherent mistrust of physicians and the public towards these innovations. Protein-activated materials are currently used in clinical practice. However, they still have drawbacks that limit their widespread use. Gene-activated materials have more advantages. They are easier to manufacture and store, and they enable local physiological protein synthesis through mechanisms present in the cell.

Although there is already a clinically approved gene-activated bone graft substitute for use in dentistry, there are still no materials that have a significant osteoinductive effect. Recent developments, physician demand and market trends all suggest that such substitutes will be available in the near future.

Dentistry and oral and maxillofacial surgery are likely to be the first clinical areas in which gene-activated materials will be tested and widely used. It will have a major impact on the future of dental care for patients with maxillofacial defects.

## 6 Ethical clearance

Not applicable, as this research was not using any animal model.

## Funding

This research was funded by the Russian Science Foundation, grant number 22-15-00425.

## CRediT authorship contribution statement

**A.V.V.:** Conceptualization, Data curation, Methodology, Visualization, Writing—original draft preparation. **V.S.K.:** Conceptualization, Investigation, Writing—original draft preparation, Visualization. **A.A.K.:** Funding acquisition, Writing—review and editing, Project administration. **I.A.N.:** Writing—review and editing. **T.B.B.:** Writing—review and editing. **D.V.G.:** Writing—review and editing. **V.K.P.:** Writing—review and editing. **D.V.G.:** Supervision. **V.K.P.:** Supervision.
